# Supplementary material for: The fission yeast cytokinetic ring component Fic1 promotes septum formation
Source: Biol Open. 2023 May 9;12(5):bio059957. doi: 10.1242/bio.059957 (PMC10184318; doi:10.1242/bio.059957)
Supplement: Supplementary information [file biolopen-12-059957-s1.pdf]

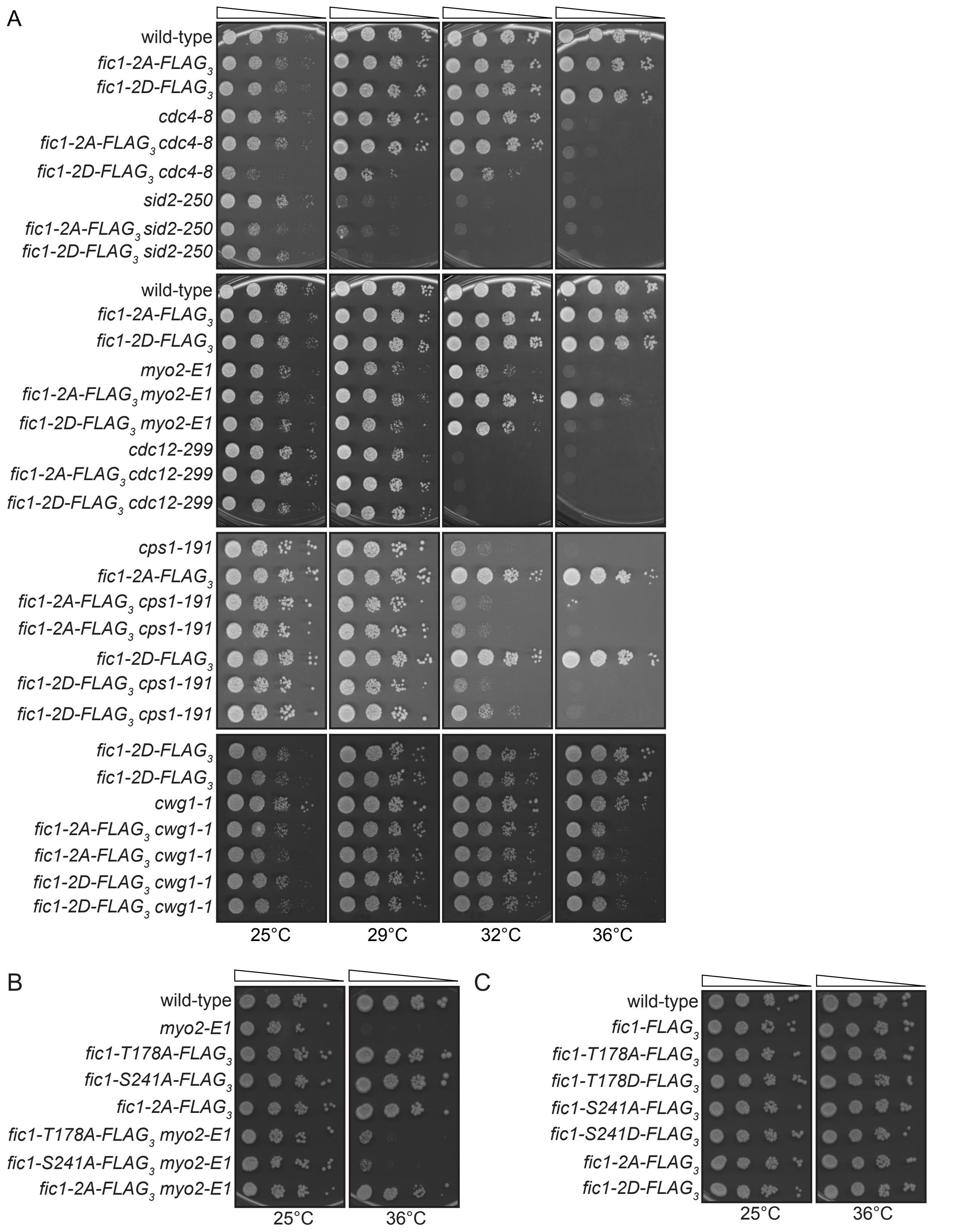

Fig. S1. Individual or single *fic1* phosphomutants are not temperature-sensitive. A-C) Ten- fold serial dilutions of the indicated strains were spotted on YE agar media and incubated at the indicated temperatures for 3-5 days.

Biology Open • Supplementary information

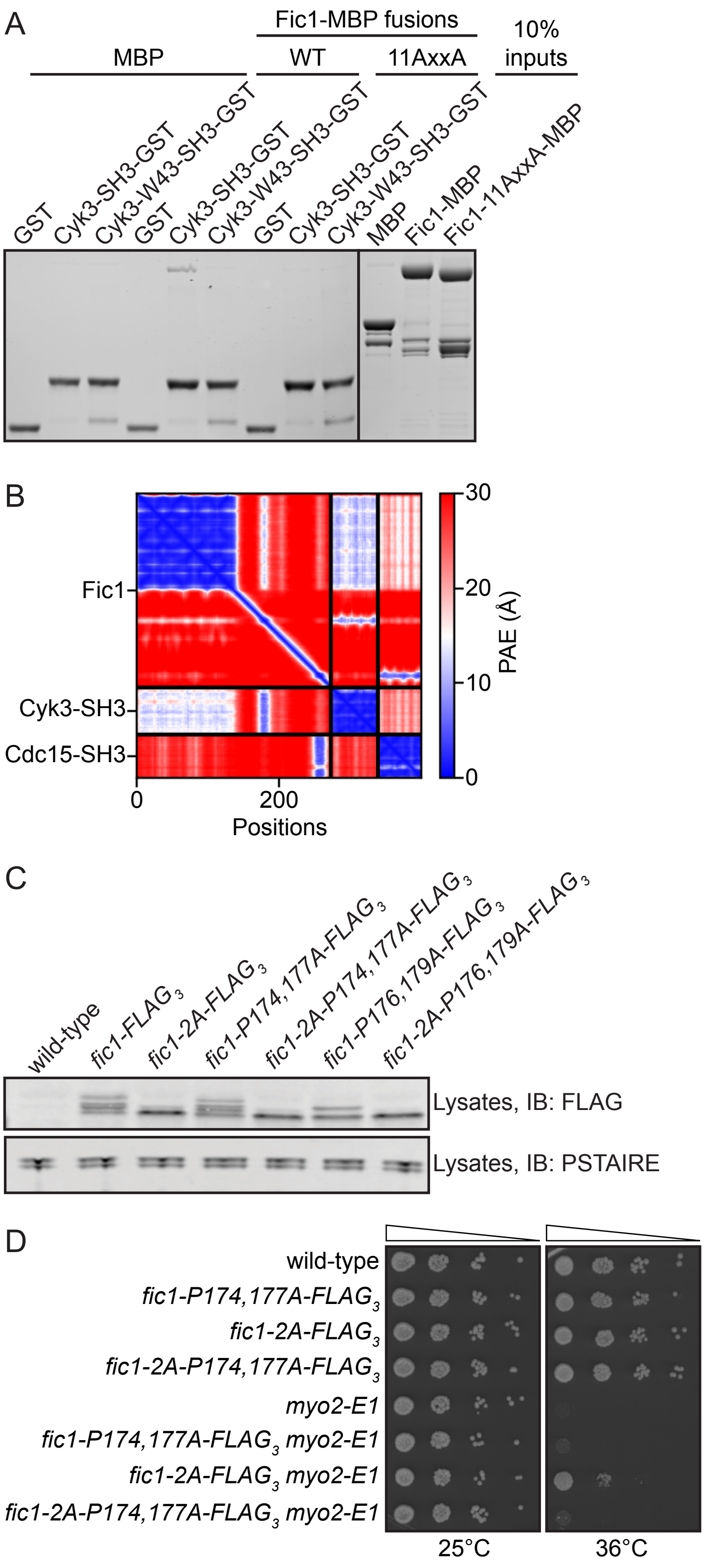

Biology Open • Supplementary information

Fig. S2. P174,177 is required for *fic1-2A*'s suppression of *myo2-E1*. A) Coomassie-stained SDS–PAGE of *in vitro* binding assays using the indicated recombinant proteins. B) The predicted aligned error (PAE) map from the molecular modeling between Fic1, Cyk3-SH3, and Cdc15-SH3. C) Lysates from cells of the indicated genotypes were immunoblotted with anti-FLAG antibody to assess Fic1-FLAG$_3$ gel mobilities and anti-CDK (PSTAIRE) antibody as a loading control. D) Ten-fold serial dilutions of the indicated strains were spotted on YE agar media and incubated at the indicated 11 temperatures for 3-5 days.

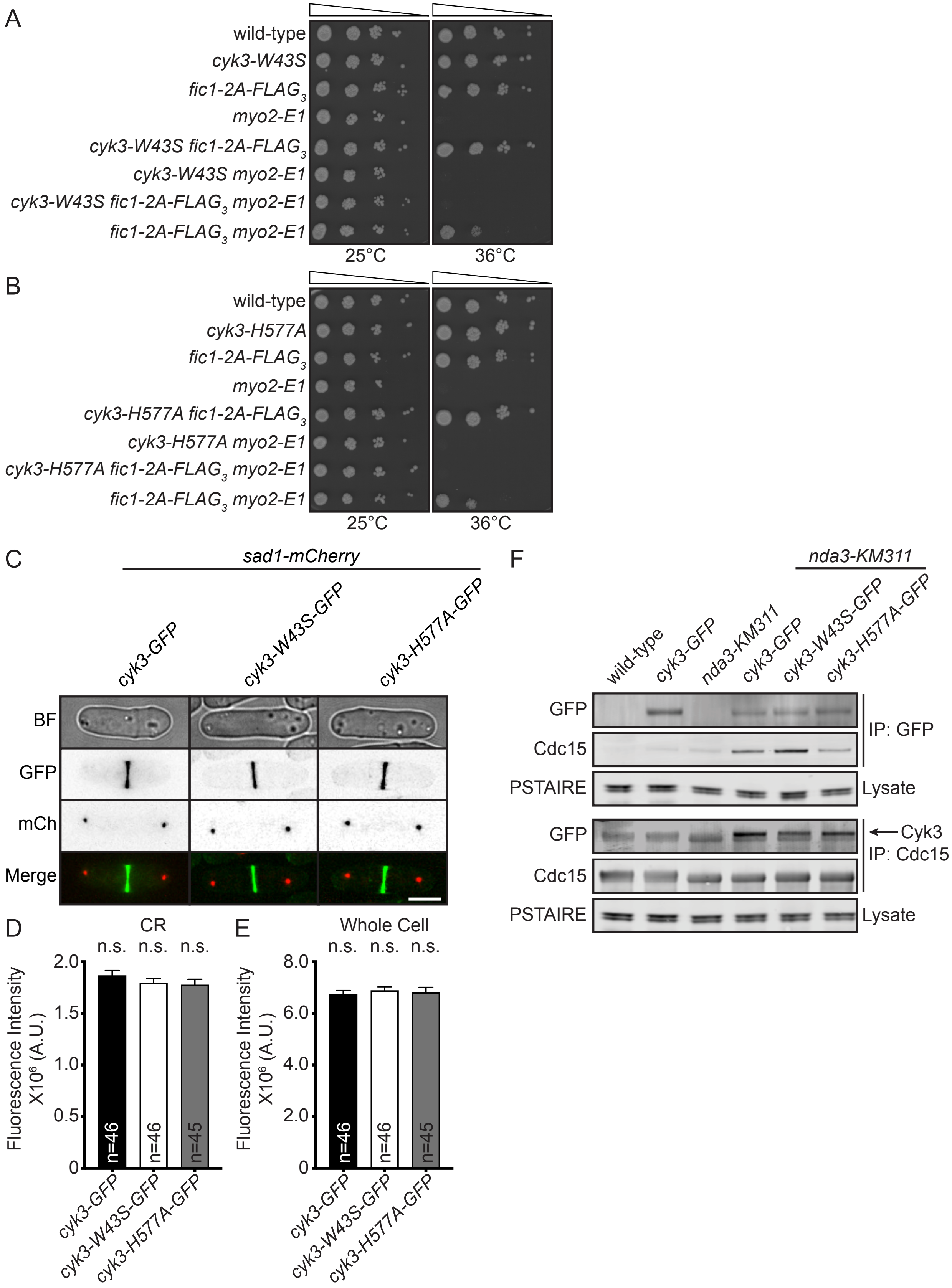

Biology Open • Supplementary information

Fig. S3. Cyk3's SH3 and transglutaminase-like domain are required for *fic1-2A's* suppression of *myo2-E1*. A and B) Ten-fold serial dilutions of the indicated strains were spotted on YE agar media and incubated at the indicated temperatures for 3-5 days. C) Live-cell bright field (BF), GFP, mCherry (mCh) and merged GFP/mCh images of cells of indicated genotypes during cytokinesis. Scale bar: 5 µm. D and E) Quantification of CR (D) and whole cell (E) fluorescence intensities for cells of indicated genotypes. Data from three trials per genotype presented as mean ± S.E.M. n.s., not significant, one-way ANOVA. F) Anti-GFP or anti-Cdc15 immunoprecipitates from cells of indicated genotypes were blotted with an anti-GFP or anti-Cdc15 antibody. Lysate samples were blotted with anti-CDK (PSTAIRE) as an input control for the immunoprecipitations. Arrow indicates Cyk3-GFP protein band.

Table S1. *S. pombe* strains used in this study

**Figure 1**		
KGY246	*ade6-M210 ura4-D18 leu1-32 h⁻*	Lab Stock
KGY11856	*fic1-T178A,S241A-FLAG$_3$:kan^R ade6-M210 ura4-D18 leu1-32 h$^+$*	Lab Stock
KGY11861	*fic1-T178D,S241D-FLAG$_3$:kan^R ade6-M210 ura4-D18 leu1-32 h$^+$*	Lab Stock
KGY2971	*myo2-E1 ade6-M216 leu1-32 ura4-D18 h$^+$*	Lab Stock
KGY1018-2	*myo2-E1 fic1-T178A,S241A-FLAG$_3$:kan^R ade6-M210 leu1-32 ura4-D18 h$^-$*	This Study
KGY15368	*myo2-E1 fic1-T178D,S241D-FLAG$_3$:kan^R ade6-M210 leu1-32 ura4-D18 h$^+$*	This Study
KGY6008	*fic1::ura4$^+$ leu1-32 ura4-D18 ade6-M21X h$^+$*	Lab Stock
KGY6665	*fic1::ura4$^+$ myo2-E1 leu1-32 ura4-D18 ade6-M21X h$^+$*	Lab Stock
KGY5305-2	*sid4-GFP:kan^R rlc1-mNeonGreen:hyg^R ade6-M210 ura4-D18 leu1-32 h$^+$*	This Study
KGY5659-2	*fic1::ura4$^+$ sid4-GFP:kan^R rlc1-mNeonGreen:hyg^R ade6-M21X ura4-D18 leu1-32 h$^-$*	This Study
KGY5602-2	*fic1-T178A,S241A-FLAG$_3$:kan^R sid4-GFP:kan^R rlc1-mNeonGreen:hyg^R ade6-M210 ura4-D18 leu1-32 h$^+$*	This Study
KGY5334-2	*fic1-T178D,S241D-FLAG$_3$:kan^R sid4-GFP:kan^R rlc1-mNeonGreen:hyg^R ade6-M210 ura4-D18 leu1-32 h$^+$*	This Study
**Figure 2**		
KGY2152-2	*act1:LactC2-GFP:nat^R sad1-GFP:kan^R ade6-M210 ura4-D18 leu1-32 h$^+$*	(Curto et al., 2014)
KGY4102-2	*fic1-T178A,S241A-FLAG$_3$:kan^R act1:LactC2-GFP:nat^R sad1-GFP:kan^R ade6-M210 ura4-D18 leu1-32 h$^+$*	This Study
KGY3655-2	*myo2-E1 act1:LactC2-GFP:nat^R sad1-GFP:kan^R ade6-M210 ura4-D18 leu1-32 h$^+$*	This Study
KGY4138-2	*fic1-T178A,S241A-FLAG$_3$:kan^R myo2-E1 act1:LactC2-GFP:nat^R sad1-GFP:kan^R ade6-M210 ura4-D18 leu1-32 h$^+$*	This Study
**Figure 3**		
KGY11876	*fic1-P257A-FLAG$_3$:kan^R leu1-32 ura4-D18 ade6-M21X h$^+$*	Lab stock
KGY11934	*fic1-T178A,S241A,P257A-FLAG$_3$:kan^R leu1-32 ura4-D18 ade6-M21X h$^+$*	This Study
KGY3373-2	*fic1-P257A-FLAG$_3$:kan^R myo2-E1 leu1-32 ura4-D18 ade6-M21X h$^-$*	This Study
KGY19546	*fic1-T178A,S241A,P257A-FLAG$_3$:kan^R myo2-E1 leu1-32 ura4-D18 ade6-M21X h$^-$*	This Study
KGY7490	*cyk3::ura$^+$ ade6-M21X leu1-32 ura4-D18 h$^-$*	Lab stock
KGY15446	*cyk3::ura4$^+$ fic1-T178,S241A-FLAG$_3$:kan^R ade6-M21X leu1-32 ura4-D18 h$^+$*	This Study
KGY7635	*cyk3::ura4$^+$ myo2-E1 ade6-M21X leu1-32 ura4-D18 h$^-$*	This Study

Biology Open • Supplementary information

KGY15443	*cyk3::ura4*$^{+}$ *fic1-T178,S241A-FLAG*$_{3}$*:kan*^{R} *myo2-E1 ade6-M21X leu1-32 ura4-D18 h*$^{+}$	This Study
KGY3873-2	*cps1-191 fic1-FLAG*$_{3}$*:kan*^{R} *chs2-V5*$_{3}$*:hyg*^{R} *lys1-131 ade6-M21X ura4-D18 leu1-32 h*$^{-}$	This Study
**Figure 4**		
KGY6365-2	*chs2::kan*^{R} *ade6-M210 ura4-D18 leu1-32 h*$^{-}$	This Study
KGY6580-2	*chs2::kan*^{R} *fic1-T178A,S241A-FLAG*$_{3}$*:kan*^{R} *ade6-M210 ura4-D18 leu1-32 h*$^{-}$	This Study
KGY6570-2	*chs2::kan*^{R} *myo2-E1 ade6-M210 ura4-D18 leu1-32 h*$^{+}$	This Study
KGY6571-2	*chs2::kan*^{R} *fic1-T178A,S241A-FLAG*$_{3}$*:kan*^{R} *myo2-E1 ade6-M210 ura4-D18 leu1-32 h*$^{+}$	This Study
KGY6434-2	*fic1::ura4*$^{+}$ *chs2::kan*^{R} *leu1-32 ura4-D18 ade6-M21X h*$^{+}$	This Study
KGY15954	*fic1::ura4*$^{+}$ *cyk3::ura4*$^{+}$ *leu1-32 ura4-D18 ade6-M21X h*$^{-}$	Lab stock
KGY6930-2	*chs2::kan*^{R} *cyk3::ura4*$^{+}$ *ade6-M21X leu1-32 ura4-D18 h*$^{-}$	This Study
KGY7101-2	*chs2::kan*^{R} *cyk3::ura4*$^{+}$ *fic1::ura4*$^{+}$ *ade6-M21X leu1-32 ura4-D18 h*$^{-}$	This Study
**Supplemental Figures**		
**Figure S1**		
KGY441	*cdc4-8 ade6-M210 ura4-D18 h*$^{+}$	Lab Stock
KGY15363	*fic1-T178A,S241A-FLAG*$_{3}$*:kan*^{R} *cdc4-8 ade6-M21X ura4-D18 h*$^{-}$	This Stock
KGY15364	*fic1-T178A,S241D-FLAG*$_{3}$*:kan*^{R} *cdc4-8 ade6-M21X ura4-D18 h*$^{+}$	This Stock
KGY1105	*sid2-250 ade6-M21X ura4-D18 leu1-32 h*$^{-}$	Lab Stock
KGY15374	*fic1-T178,S241A-FLAG*$_{3}$*:kan*^{R} *sid2-250 ade6-M210 ura4-D18 leu1-32 h*$^{-}$	This Study
KGY15375	*fic1-T178,S241D-FLAG*$_{3}$*:kan*^{R} *sid2-250 ade6-M210 ura4-D18 leu1-32 h*$^{-}$	This Study
KGY748	*cdc12-299 h*$^{-}$	Lab Stock
KGY15365	*fic1-T178,S241A-FLAG*$_{3}$*:kan*^{R} *cdc12-299 leu1-32 ura4-D18 h*$^{+}$	This Study
KGY15366	*fic1-T178,S241D-FLAG*$_{3}$*:kan*^{R} *cdc12-299 299 leu1-32 ura4-D18 h*$^{+}$	This Study
KGY17207	*cps1-191 ura4-D18 leu1-32 ade6-M210 lys1-131 h*$^{+}$	Lab Stock
KGY17771	*cps1-191 fic1-T178A,S241A-FLAG*$_{3}$*:kan*^{R} *ura4-D18 ade6-M21X h*$^{-}$	This Study
KGY17773	*cps1-191 fic1-T178D,S241D-FLAG*$_{3}$*:kan*^{R} *ura4-D18 ade6-M21X h*$^{-}$	This Study
KGY11106	*cwg1-1 leu1-32 h*$^{-}$	Lab Stock
KGY17681	*cwg1-1 fic1-T178A,S241A-FLAG*$_{3}$*:kan*^{R} *ade6-M21X ura4-D18 leu1-32 h*$^{-}$	This Study
KGY17683	*cwg1-1 (bgs4 ts) fic1-T178D,S241D-FLAG*$_{3}$*:kan*^{R} *ade6-M21X ura4-D18 leu1-32 h*$^{-}$	This Study
KGY19522	*fic1-T178A-FLAG*$_{3}$*:kan*^{R} *ade6-M210 ura4-D18 leu1-32 h*$^{+}$	Lab Stock

KGY19523	*fic1-S241A-FLAG$_3$:kan^R ade6-M210 ura4-D18 leu1-32 h$^+$*	Lab Stock
KGY7615-2	*fic1-T178A-FLAG$_3$:kan^R myo2-E1 ade6-M210 ura4-D18 leu1-32 h$^-$*	This Study
KGY7628-2	*fic1-S241A-FLAG$_3$:kan^R myo2-E1 ade6-M210 ura4-D18 leu1-32 h$^-$*	This Study
KGY6288	*fic1-FLAG$_3$:kan^R ade6-M216 leu1-32 ura4-D18 h$^-$*	Lab stock
KGY11908	*fic1-T178D-FLAG$_3$:kan^R ade6-M210 ura4-D18 leu1-32 h$^+$*	Lab stock
KGY11854	*fic1-S241D-FLAG$_3$:kan^R leu1-32 ura4-D18 ade6-M21X h$^+$*	Lab stock
**Figure S2**		
KGY4705-2	*fic1-P174,177A-FLAG$_3$:kan^R leu1-32 ura4-D18 ade6-M21X h$^+$*	This Study
KGY4774-2	*fic1-T178A,S241A-P174,177A-FLAG$_3$:kan^R leu1-32 ura4-D18 ade6-M21X h$^+$*	This Study
KGY4706-2	*fic1-P176,179A-FLAG$_3$:kan^R leu1-32 ura4-D18 ade6-M21X h$^+$*	This Study
KGY4708-2	*fic1-T178A,S241A-P176,179A-FLAG$_3$:kan^R leu1-32 ura4-D18 ade6-M21X h$^+$*	This Study
KGY4717-2	*fic1-P174,177A-FLAG$_3$:kan^R myo2-E1 leu1-32 ura4-D18 ade6-M21X h$^+$*	This Study
KGY4852-2	*fic1-T178A,S241A-P174,177A-FLAG$_3$:kan^R myo2-E1 leu1-32 ura4-D18 ade6-M21X h$^-$*	This Study
**Figure S3**		
KGY899-2	*cyk3-W43S ade6-M21X leu1-32 ura4-D18 h$^-$*	This Study
KGY2086-2	*cyk3-W43S fic1-T178A,S241A-FLAG$_3$:kan^R ade6-M21X leu1-32 ura4-D18 h$^+$*	This Study
KGY2141-2	*cyk3-W43S myo2-E1 ade6-M21X leu1-32 ura4-D18 h$^-$*	This Study
KGY2505-3	*cyk3-W43S fic1-T178A,S241A-FLAG$_3$:kan^R myo2-E1 ade6-M21X leu1-32 ura4-D18 h$^-$*	This Study
KGY1886-2	*cyk3-H577A ade6-M21X leu1-32 ura4-D18 h$^-$*	This Study
KGY2120-2	*cyk3-H577A fic1-T178A,S241A-FLAG$_3$:kan^R ade6-M21X leu1-32 ura4-D18 h$^+$*	This Study
KGY2161-2	*cyk3-H577A myo2-E1 ade6-M21X leu1-32 ura4-D18 h$^-$*	This Study
KGY2559-2	*cyk3-H577A fic1-T178A,S241A-FLAG$_3$:kan^R myo2-E1 ade6-M21X leu1-32 ura4-D18 h$^-$*	This Study
KGY6897	*cyk3-FLAG$_3$:kan^R ade6-M21X leu1-32 ura4-D18 h$^-$*	Lab Stock
KGY2460-2	*cyk3-GFP:kan^R sad1-mCherry:nat^R ade6-M210 ura4-D18 leu1-32 h$^-$*	This Study
KGY2362-2	*cyk3-W43S-GFP:kan^R sad1-mCherry:nat^R ade6-M21X leu1-32 ura4-D18 h$^-$*	This Study
KGY2364-2	*cyk3-H577A-GFP:kan^R sad1-mCherry:nat^R ade6-M21X leu1-32 ura4-D18 h$^-$*	This Study
KGY2202-3	*cyk3-GFP:kan^R ade6-M210 ura4-D18 leu1-32 h$^-$*	Lab Stock
KGY56	*nda3-km311 leu1-32 h$^+$*	Lab Stock

Biology Open • Supplementary information

KGY2890-2	*cyk3-GFP:kan^R nda3-km311 ade6-M210 ura4-D18 leu1-32 h$^-$*	This Study
KGY2895-2	*cyk3-W43S-GFP:kan^R nda3-km311 ura4-D18 leu1-32 h$^-$*	This Study
KGY2892-2	*cyk3-H577A-GFP:kan^R nda3-km311 ade6-M210 ura4-D18 leu1-32 h$^-$*	This Study

Biology Open • Supplementary information
